# PARTNER 2A & SAPIEN 3: TAVI for intermediate risk patients

**DOI:** 10.21542/gcsp.2016.33

**Published:** 2016-12-30

**Authors:** Ahmed ElGuindy

**Affiliations:** Department of Cardiology, Aswan Heart Centre

## Abstract

Transcatheter aortic valve implantation (TAVI) is currently indicated for patients with severe symptomatic aortic stenosis who are unfit for surgery or when the surgical risk is high. Thanks to the increasing experience of surgeons, better patient selection, and substantial improvements in device technology, the procedure is now being performed with excellent outcomes and a progressively lower rate of complications. As a result, the cut-off threshold to implant a transcatheter valve is gradually moving toward lower risk patients. However, this is not supported by strong evidence from rigorous large clinical trials. The PARTNER 2A and SAPIEN 3 trials were conducted to address this gap in our knowledge.

## Introduction

Transcatheter aortic valve implantation (TAVI), also known as transcathter aortic valve replacement (TAVR), is currently indicated for patients with severe symptomatic aortic stenosis and a prohibitive risk for surgery, or in patients with high risk for surgical aortic valve replacement (SAVR). Since Alain Cribier performed the first successful implant in 2002,^1^ progress in this field has been truly remarkable. With surgeons gaining more experience in both case selection and technical aspects of the procedure, and with the tremendous improvements in device technology, TAVI has become a safer procedure with more predictable and reproducible outcomes. The latest generation of commercially available valves can be delivered via sheaths as small as 14F. Greater than mild paravalvular leaks, an Achilles heal of the procedure, has now dropped to less than 3% in many series. The procedure itself is now being performed under conscious sedation in many centers around the world, and the overall length of hospital stay continues to decline steadily.

These substantial advances, coupled with encouraging outcomes from small studies^2–5^ have led to a worldwide trend to use TAVI in low and/or intermediate risk patients. However, such practice is not supported by solid evidence from large clinical trials. The PARTNER 2A and SAPIEN 3 trials, two of the largest TAVI trials conducted to date, were conducted to address this knowledge gap using two different generations of valves that are currently being used in clinical practice – the Sapien XT and Sapien 3 respectively. Results of both trials were recently presented at the ACC.16 Scientific Sessions and were published simultaneously in the *New England Journal of Medicine* and *The Lancet*. ^6,7^

## PARTNER 2A

### Trial design

PARTNER 2A was in fact two parallel prospective randomized multicenter trials using the same transcatheter valve (Sapien XT - Edwards Lifesciences, USA). The study enrolled 2,032 patients with severe symptomatic aortic stenosis and with an intermediate risk for SAVR. Patients were considered to be intermediate risk based on a detailed assessment by a multidisciplinary heart team. Intermediate risk was defined as a Society of Thoracic Surgeons (STS) score of 4–8%. Patients with an STS score of less than 4% were also enrolled if they were judged by the heart team to be at a higher risk because of other conditions that are not represented in the STS risk model. Exclusion criteria included recent myocardial infarction, bicuspid (or unicuspid) aortic valves, predominant aortic regurgitation, preexisting artificial valve in any position, complex coronary disease (unprotected left main disease or Syntax score >32), left ventricular ejection fraction <20%, active upper GI bleeding within 90 days prior to the procedure, contraindication to anticoagulant therapy, native aortic annulus size <18mm or >27mm, severe renal insufficiency (creatinine >3 mg/dL) and/or renal replacement therapy, and patients with an estimated life expectancy <24 months due to other comorbidities.

Eligible patients were stratified into two cohorts after evaluating the suitable access route (transfemoral or transthoracic), and were then randomly assigned in a 1:1 ratio to undergo either transcatheter or surgical aortic valve replacement. Among the 2,032 patients who underwent randomization, 1,011 were assigned to TAVI and 1,021 to SAVR. 1,550 patients (76.3%) were judged to be suitable for transfemoral TAVI. The mean STS score was 5.8% in both groups, with 6.7% of the patients having an STS score of less than 4%, and 12% having a score that was greater than 8%.

The study’s primary endpoint was the composite of death from any cause or disabling stroke (score ≥2 on the modified Rankin scale) at 2 years. Secondary endpoints included major vascular complications, life-threatening bleeding, acute kidney injury, new-onset atrial fibrillation, paravalvular regurgitation, and permanent pacemaker implantation. The study was powered to detect differences by both intention-to-treat and as-treated analyses.

### Results

The composite primary endpoint of death from any cause or disabling stroke did not differ between both groups (hazard ratio in the TAVI group 0.89; 95% confidence interval [CI], 0.73–1.09; *p* = 0.25). This held true in both the intention-to-treat and as-treated analyses. The risk ratio at 2 years for the primary endpoint in the TAVI group was 0.92 (95% CI, 0.77–1.09) compared to the SAVR group. This risk ratio met the prespecified criterion for noninferiority (*p* = 0.001). 18 patients (10 in the TAVI group and 8 in the SAVR group) died during the procedure or within 3 days afterwards. The incidence of the individual components of the primary endpoint were also similar between both groups (death from any cause; 16.7% with TAVI and 18% with SAVR, disabling stroke; 6.2% with TAVI and 6.4% with SAVR).

In the transfemoral-access cohort, TAVI resulted in a borderline significant reduction in death from any cause or disabling stroke compared to SAVR (hazard ratio, 0.79; 95% CI, 0.62–1.00; *p* = 0.05). However, there was no significant between-group difference in the transthoracic-access cohort (hazard ratio, 1.21; 95% CI, 0.84–1.74; *p* = 0.31). It is important to mention that the transfemoral and transthoracic cohorts were pre-specified but were not powered to detect differences in the primary endpoint between the TAVI and SAVR groups.

With respect to key secondary endpoints, major vascular complications were more frequent in the TAVI group compared to the SAVR group (7.9% vs. 5% respectively; *p* = 0.008). The incidence of greater-than-mild paravalvular regurgitation was also higher after TAVI (3.7% vs. 0.2%; *p* < 0.001). On the other hand, TAVI was associated with significantly less life threatening bleeding (10.4% vs. 43.4%; *p* < 0.001), acute kidney injury (1.3% vs. 3.1%, *p* = 0.006), and new-onset atrial fibrillation (9.1% vs. 26.4%, *p* < 0.001). The need for new permanent pacemakers within 30 days of the procedure did not differ between both groups (8.5% vs. 6.9% for TAVI and SAVR respectively; *p* = 0.17). Furthermore, patients in the TAVI group had a significantly shorter index hospitalization compared to those in the SAVR group (median 6 vs. 9 days; *p* < 0.001).

## SAPIEN 3

### Trial design

SAPIEN 3 was an observational study in which 1,077 intermediate risk patients with severe symptomatic aortic stenosis received TAVI using the third generation Sapien 3 valve (Edwards Lifesciences). The results at one year were compared to the those in the surgical cohort of the PARTNER 2A trial using an innovative pre-specified propensity-score analysis to control for the different patient characteristics. The analysis incorporated 22 pre-specified baseline characteristics that were factored through a logistic regression model. Patients were stratified into 5 quintiles which allowed for cohort matching – rather than patient matching – which made it possible to capture all available data for the analysis, rather than end with a limited number of patients in each arm as the case usually is with conventional propensity matching. This design made it possible to evaluate clinical outcomes with the latest generation Sapien 3 valve – which only became available after the PARTNER 2A trial had started – in a comparative (but non-randomized) manner.

Inclusion and exclusion criteria were identical to PARTNER 2A. The primary endpoint however was different; being the composite of death from any cause, all strokes, and incidence of moderate or severe aortic regurgitation in SAPIEN 3.

Compared to the previous valve, the newer valve has a number of important design improvements including improved geometry of the trileaflet bovine pericardial valve; different cobalt alloy frame with more open cells on its aortic (outflow) portion to improve coronary blood flow; a second fine-adjustment wheel and enhanced flexion of the delivery system for more precise positioning; and perhaps most importantly, an external polyethylene terephthalate fabric skirt (sealing cuff) sewn to the bottom portion of the frame to reduce paravalvular leaks. The valve also has a lower-profile delivery system which can be inserted through 14–16 French sheaths.

### Results

Most baseline characteristics were similar between both groups. Patients in the SAPIEN 3 group had more frequent oxygen-dependent chronic obstructive lung disease. Patients in the PARTNER 2A surgery group had mildly higher mean STS scores (5.4% vs. 5.2%; *p* = 0.0004), lower left ventricular ejection fraction (55.4% vs. 58.5%; *p* < 0.0001), and more frequent moderate or severe mitral regurgitation (18% vs. 9%; *p* < 0.0001).

Compared to the surgical group, the TAVI group had a lower incidence of the composite primary endpoint of death from any cause, all strokes, and incidence of moderate or severe aortic regurgitation in each of the five quintiles (ranging from −14.5% in quintile 1 to −4.3% in quintile 5). The pooled difference in the incidence of the primary endpoint across all quintiles was significantly lower in the TAVI group (−9.2%; 90% CI −12.4 to −6 in favor of TAVI). With regards to the individual components of the primary endpoint, mortality at one year and stroke were lower with TAVI compared to surgery (−5.2%; CI −8 to −2.4; *p* < 0.0001 and −3.5%, –CI 5.9 to −1.1; *p* = 0.0038). On the other hand, surgery was superior to TAVI with regards to moderate or severe aortic regurgitation (1.2%, CI 0.2 to 2.2; *p* = 0.0149).

## Discussion

At a time where enthusiasm (by both patients and physicians) for TAVI is on the rise, PARTNER 2A and SAPIEN 3 both provide badly-needed data that helps close the gap between evidence and practice. Both studies provide reasonably reassuring evidence that TAVI can be offered to intermediate risk patients with outcomes that are at least comparable to surgery.

While the non-randomized design of SAPIEN 3 carries inherent limitations and the risk of unmeasured confounders, the robust propensity matching methodology, identical inclusion and exclusion criteria, the same clinical event committee, near-identical clinical sites and operators as well as the same core echocardiography laboratory make it very unlikely for its key findings to be a “play of chance”. In addition, results from SAPIEN 3 emphasize earlier findings from smaller studies, showing an excellent safety profile of the newer generation valves with marked reduction in moderate or severe paravalvular leaks, dropping to only 2% in SAPIEN 3. Although the incidence of mild paravalvular leaks was high in both studies (approximately 40%), this did not have an effect on clinical outcomes. This is in contrast to findings from the original PARTNER trial where mild paravalvular regurgitation was associated with worse survival compared to none/trace regurgitation. The improved delivery profile of the newer valve (SAPIEN 3) also allowed for a transfemoral route in 88% of patients in SAPIEN 3 compared to 76% in PARTNER 2 with numerically less major vascular complications (6.1% vs. 7.9% respectively).

Both studies continue to strengthen a culture that has been inherent to TAVI since its emergence; the Heart Team approach. This multidisciplinary approach continues to be an asset to and a major achievement of TAVI, and has been critical to the overall success of the procedure to date. Interestingly, results of both trials were presented by cardiac surgeons at the ACC scientific meeting.

It is important to understand the limitations of both studies in terms of the patient population enrolled. Both studies enrolled *elderly* patients; with a mean age of 82 years. Both trials excluded patients with bicuspid aortic valves (BAV), who may represent a substantial percentage of younger patients with severe aortic stenosis. While BAV per se is not an absolute contraindication to TAVI, and in fact many centers around the world are implanting transcatheter valves in this group of patients, the limitations of TAVI in this setting are well recognized.^8–10^ Paravalvular leaks in particular seem to be more common with BAV owing to a number of morphological features of the cusps, annulus and aortic root in addition to the pattern and extent of calcification.^8^

Furthermore, accurate measurement of the annulus in patients with BAV, and hence selection of the appropriate valve size, can be challenging. Patients with complex coronary disease and those with severe left ventricular systolic dysfunction were also excluded from both trials. It is still unknown whether TAVI can be comparable to SAVR in these patient populations. Recent concerns about leaflet thrombosis and reduced mobility,^11^ as well as concerns about the long-term durability of the valve cusps and platform will also need to be addressed before TAVI becomes the treatment-of-choice for severe aortic stenosis in intermediate risk patients, particularly younger ones.

Finally, with the current valve prices, it remains to be seen whether TAVI will be cost effective compared to surgery in patients with less surgical risk and fewer comorbidities.

## What have we learned?

Results from PARTNER 2A and SAPIEN 3 strongly suggest that TAVI is a suitable alternative to SAVR in intermediate risk patients with severe symptomatic aortic stenosis. The results also suggest that transfemoral TAVI and the use of the third generation Sapien 3 valve is associated with better outcomes compared to SAVR. As the TAVI landscape moves toward younger patients with less comorbidities and longer life expectancy, it becomes crucial to evaluate the rate of valve degeneration over longer periods of time. These results also warrant further studies to evaluate the role of TAVI in low-risk patients, which represent more than 75% of patients undergoing aortic valve replacement. There are currently two ongoing trials in the United States alone that are investigating the outcomes of TAVI in this subset of patients.
